# Atividade física e HDL-C: Existem Diferenças entre os Sexos no Efeito Dose-resposta?

**DOI:** 10.36660/abc.20210551

**Published:** 2021-09-01

**Affiliations:** 1 Universidade Federal do Rio Grande do Sul Programa de Pós-graduação em Cardiologia e Ciências Cardiovasculares Hospital de Clínicas de Porto Alegre Porto Alegre RS Brasil Programa de Pós-graduação em Cardiologia e Ciências Cardiovasculares – Universidade Federal do Rio Grande do Sul (UFRGS), Hospital de Clínicas de Porto Alegre (HCPA), Porto Alegre, RS – Brasil; 2 Grupo de Pesquisa em Cardiologia do Exercício Porto Alegre RS Brasil Grupo de Pesquisa em Cardiologia do Exercício (CardioEx) – UFRGS, HCPA, Porto Alegre, RS – Brasil; 3 Universidade de São Paulo Unidade Clínica de Lípides do Instituto do Coração (InCor) Faculdade de Medicina São Paulo SP Brasil Unidade Clínica de Lípides do Instituto do Coração (InCor) – Hospital das Clínicas da Faculdade de Medicina da Universidade de São Paulo (HC-FMUSP), São Paulo, SP – Brasil; 4 Hospital Israelita Albert Einstein São Paulo SP Brasil Hospital Israelita Albert Einstein, São Paulo, SP – Brasil

**Keywords:** HDL-colesterol, Exercício Físico, Doenças Cardiovasculares

## HDL: funções básicas e controvérsias cardiovasculares

As lipoproteínas de alta densidade (HDL) são um grupo heterogêneo de partículas^1^ cuja função principal é a promoção do transporte reverso do colesterol.^2^ Evidências geradas durante décadas nos ensinaram que ter concentrações plasmáticas baixas do colesterol carregado pelas HDL (HDL-C) parece não ser saudável. Ainda em 1964, Kannel et al.^3^ demonstraram – por meio de uma análise da coorte de Framingham – uma forte relação inversa entre níveis plasmáticos de HDL-C e doença aterosclerótica cardiovascular. Entretanto, este paradigma tem se modificado em anos mais recentes. O papel causal da HDL na redução do risco cardiovascular foi contestado por estudos de randomização mendeliana, que falharam em demonstrar redução do risco de infarto do miocárdio em indivíduos com algumas variantes genéticas que aumentavam o HDL-C.^4^^,^^5^ Além disso, a elevação do HDL-C obtida por via farmacológica com os inibidores da proteína transportadora de ésteres de colesterol (CETP) também não levou à redução em eventos cardiovasculares.^6^ Finalmente, estudos que mostram associação de maior mortalidade com valores altos e baixos de HDL-C colocaram mais dúvidas nessa situação.^7^ Assim, atualmente, entende-se que a proteção cardiovascular das HDL é, no mínimo, controversa, e que o HDL-C parece atuar muito mais como um marcador de risco cardiovascular do que propriamente um fator causal.

## HDL-C e exercício físico/atividade física em homens e mulheres

Apesar das controvérsias, perda de peso, cessação de fumo e exercício físico/atividade física (AF) continuam a ser recomendados para indivíduos com HDL-C baixo.^8^^–^^10^ Contudo, o conhecimento acerca da dose-resposta sobre exercício físico e AF sobre o HDL-C em homens e mulheres é um tanto escasso e controverso. De qualquer modo, mudanças nas concentrações plasmáticas de HDL-C por meio de treinamento físico parecem estar intimamente relacionadas a sexo (homens e mulheres), intensidade, frequência e duração do treinamento.

Em uma revisão sistemática que englobou 23 metanálises de ensaios clínicos randomizados, as quais avaliaram o papel do exercício físico nos níveis de HDL-C, foi encontrado um aumento que variou de 0,27 a 5,41mg/dL; entretanto, houve uma grande variabilidade nos tipos, duração e intensidade dos exercícios, bem como das populações nas metanálises incluídas, além de um baixo nível de confiança.^11^ Isso aponta para a necessidade de mais estudos bem delineados nesta área do conhecimento.

Ainda nos anos 1990, Kokkinos et al.^12^ já sugeriam que mudanças favoráveis no HDL-C poderiam estar associadas a uma maior intensidade de treinamento. Esses pesquisadores aplicaram um questionário em mais de 2.900 homens saudáveis não fumantes e com média de idade de 43 anos. Foi observado aumento de 7% no HDL-C daqueles indivíduos que corriam entre 100 e 120 minutos/semana por aproximadamente 11 a 22 km de distância/semana, quando comparados aos sedentários de características semelhantes. Sugere-se que o sexo, a duração do exercício e a intensidade estão entre os fatores responsáveis pelas diferenças nas respostas das lipoproteínas ao exercício. Em outro estudo, com população da coorte ATTICA (n = 2.772, média de idade de 45 anos), foi encontrada associação entre AF e aumento nos níveis de HDL-C em mulheres, mas não nos homens, após ajuste para variáveis como idade, tabagismo e índice de massa corporal (IMC).^13^ Já na coorte de Jan et al.^14^ com aplicação de questionário sobre níveis de exercício, durações abaixo ou acima de 2,5 horas/semana correlacionaram-se com aumento do HDL-C em ambos os sexos, sendo mais pronunciadas nos homens do que nas mulheres em ambas as durações de treinamento.

Com relação ao sedentarismo, as evidências apontam para dados preocupantes quanto à inatividade física no Brasil.^15^ Ao longo de quase 30 anos (1990-2017), os brasileiros demonstraram um risco de exposição à inatividade física de 59%, tanto em 1990 quanto em 2017; além disso, aproximadamente 22.500 e 32.400 mortes por todas as causas foram atribuíveis à inatividade física nesses anos, respectivamente.

Nesta edição dos Arquivos, Pitanga et al.^16^ avaliaram a associação na dose-resposta do tempo fisicamente ativo com o HDL-C, e se havia diferenças comparando-se os sexos em uma análise transversal dos participantes do Estudo Longitudinal de Saúde do Adulto (ELSA-Brasil). No total, 13.931 participantes (idade média: 55 anos, 45% homens) foram analisados. Entre os homens, quase metade era tabagista, enquanto, entre as mulheres, esse percentual foi de 36%. Aproximadamente ¼ dos homens e 30% das mulheres eram obesos. Valores de HDL-C ≥ 60 mg/dL foram considerados como elevados. Apenas 14,5% dos homens apresentaram HDL-C nesse patamar, em comparação com aproximadamente 43% das mulheres. Vale lembrar que, apesar de um maior percentual de mulheres apresentar HDL-C elevado, mais de 36% delas eram fumantes, fato que poderia alterar a funcionalidade das HDL.^17^

O estudo de Pitanga et al.^16^ também corrobora com dados prévios^15^ e fortalece a necessidade de uma vida menos sedentária entre os brasileiros. Nesse sentido, os níveis de AF no tempo livre foram medidos utilizando-se o questionário IPAQ (do inglês, *International Physical Activity Questionnaire*). Um baixo percentual, tanto de homens quanto de mulheres, foi considerado como ativo ou muito ativo (27,6% *vs.* 25,6%; 20,1% *vs.* 12,0%, respectivamente); mais de 30% dos homens e 44% das mulheres foram classificados como fisicamente inativos. Os pesquisadores encontraram um percentual 38% e 41% maior na chance de alcançar níveis altos de HDL-C nos homens e mulheres considerados ativos, respectivamente, quando comparados aos inativos/inativas. Nos homens, HDL-C elevado se associou com maior duração e intensidade de AF, ao passo que, nas mulheres, a AF de menor intensidade e duração também se associou a este parâmetro. Em contraste, outros autores propuseram que as mulheres, e não os homens, necessitariam de maior volume de treinamento para aumentar o HDL-C, o que poderia ser justificado por um HDL-C mais elevado nas mulheres na linha de base dos estudos em comparação aos dos homens,^12^ uma vez que o aumento do HDL-C pode ser mais pronunciado em indivíduos com níveis basais mais baixos. Infelizmente, por não haver dados basais do HDL-C dos participantes da coorte de Pitanga et al.,^16^ esta análise não se tornou possível. Por outro lado, o estudo traz hipóteses interessantes que podem ser testadas em estudos randomizados e controlados.

O estudo é limitado por sua natureza observacional, seu desenho transversal, e pela utilização de questionários de AF autorrelatados, que podem trazer certo grau de subjetividade às informações. Assim, ainda permanece o debate em relação à intensidade e volume específicos de treinamento para homens e mulheres visando a elevação do HDL-C.
