# Supplementary material for: Atividade física e HDL-C: Existem Diferenças entre os Sexos no Efeito Dose-resposta?
Source: Arq Bras Cardiol. 2021 Sep 1;117(3):501–2. [Article in Portuguese] doi: 10.36660/abc.20210551 (PMC8462943; doi:10.36660/abc.20210551)
Supplement: Translation

# Translation: Physical Activity and HDL-C: Are There Gender Differences in the Dose-response Effect?

**Authors:** Filipe Ferrari, Raul D. Santos

**Affiliations:** 1 Porto Alegre RS Brazil Programa de Pós-graduação em Cardiologia e Ciências Cardiovasculares – Universidade Federal do Rio Grande do Sul (UFRGS), Hospital de Clínicas de Porto Alegre (HCPA), Porto Alegre, RS – Brazil; 2 Porto Alegre RS Brazil Grupo de Pesquisa em Cardiologia do Exercício (CardioEx) – UFRGS, HCPA, Porto Alegre, RS – Brazil; 3 São Paulo SP Brazil Unidade Clínica de Lípides do Instituto do Coração (InCor) – Hospital das Clínicas da Faculdade de Medicina da Universidade de São Paulo (HC-FMUSP), São Paulo, SP – Brazil; 4 São Paulo SP Brazil Hospital Israelita Albert Einstein, São Paulo, SP – Brazil

**Keywords:** Cholesterol, HDL, Exercise, Cardiovascular Diseases

## HDL: Basic Functions and Cardiovascular Controversies

High-density lipoproteins (HDL) are a heterogeneous group of particles^1^ whose primary function is to promote reverse cholesterol transport.^2^ Evidence generated over decades has taught us that having low plasma concentrations of HDL-cholesterol (HDL-C) does not seem to be healthy. Also in 1964, Kannel et al.^3^ demonstrated – through an analysis of the Framingham cohort – a strong inverse relationship between plasma levels of HDL-C and atherosclerotic cardiovascular disease. However, this paradigm has changed in recent years. The causal role of HDL in cardiovascular risk reduction has been challenged by Mendelian randomization studies, which failed to demonstrate reduced risk of myocardial infarction in individuals with some genetic variants that increased HDL-C.^4^^,^^5^ In addition, the elevation of HDL-C obtained pharmacologically with cholesterol ester transporter protein (CETP) inhibitors did not lead to a reduction in cardiovascular events either.^6^ Finally, studies showing an association of higher mortality with high and low HDL-C levels raised further questions about this situation.^7^ Thus, today it is believed that the cardiovascular protection of HDL is, to say the least, controversial, and that HDL-C seems to act much more as a cardiovascular risk marker than as a causal factor.

## HDL-C and Physical Exercise/Physical Activity in Men and Women

Despite the controversies, weight loss, smoking cessation and physical exercise/physical activity (PA) continue to be recommended for individuals with low HDL-C.^8^^–^^10^ However, knowledge about the dose-response on physical exercise and PA on HDL-C in men and women is somewhat sparse and controversial. Anyway, changes in plasma HDL-C concentrations through physical training seem to be closely related to gender (men and women), intensity, frequency and duration of training.

In a systematic review that included 23 meta-analyses of randomized clinical trials, which assessed the role of physical exercise in HDL-C levels, an increase ranging from 0.27 to 5.41 mg/dL was found; however, there was great variability in exercise types, duration and intensity, and populations in the meta-analyses included, in addition to a low level of confidence.^11^ This points to the need for more well-designed studies in this field of knowledge.

Also in the 1990s, Kokkinos et al.^12^ suggested that favorable changes in HDL-C could be associated with higher training intensity. These researchers administered a questionnaire to more than 2,900 healthy men, non-smokers, with a mean age of 43. A 7% increase in HDL-C was observed in those individuals who ran 100–120 minutes/week for about 11 to 22 km/week compared to sedentary individuals with similar characteristics. It is suggested that gender, exercise duration and intensity are some of the factors responsible for differences in lipoprotein responses to exercise. In another study, with the ATTICA cohort population (n = 2,772, mean age 45), an association was found between PA and increased HDL-C levels in women, but not in men, after adjusting for variables such as age, smoking and body mass index.^13^ In the cohort of Jan et al.,^14^ with the application of a questionnaire on exercise levels, durations below or above 2.5 hours/week were correlated with an increase in HDL-C in both genders, being more pronounced in men than in women in both training durations.

Regarding a sedentary lifestyle, evidence points to worrying data related to physical inactivity in Brazil.^15^ Over almost 30 years (1990–2017), Brazilians demonstrated a 59% risk of exposure to physical inactivity, both in 1990 and in 2017; in addition, approximately 22,500 and 32,400 deaths from all causes were attributable to physical inactivity in these years, respectively.

In this issue of Arquivos, Pitanga et al.^16^ assessed the dose-response association of physically active time with HDL-C, and whether there were differences comparing genders in a cross-sectional analysis of participants in the Longitudinal Study of Adult Health (Elsa-Brazil). A total of 13,931 participants (average age: 55, 45% men) were analyzed. Among men, almost half were smokers, while among women this percentage was 36%. Approximately ¼ of men and 30% of women were obese. HDL-C ≥ 60 mg/dL levels were considered high. Only 14.5% of men had HDL-C at this level, compared to approximately 43% of women. It is worth noting that, despite a higher percentage of women with high HDL-C, more than 36% of them were smokers, a fact that could change the functionality of HDL.^17^

The study by Pitanga et al.^16^ also corroborates previous data^15^ and strengthens the need for a less sedentary life among Brazilians. In this regard, PA levels during free time were measured using the International Physical Activity Questionnaire (IPAQ). A low percentage of both men and women was considered active or very active (27.6% vs. 25.6%; 20.1% vs. 12.0%, respectively); more than 30% of men and 44% of women were classified as physically inactive. The researchers found a 38% and 41% higher percentage in the chances of achieving high levels of HDL-C in active men and women, respectively, compared to inactive men and women. In men, high HDL-C was associated with greater duration and intensity of PA, while in women, PA of smaller intensity and shorter duration was also associated with this parameter. In contrast, other authors proposed that women, rather than men, would need more training to increase HDL-C, which could be justified by a higher HDL-C among women at the baseline of studies compared to men,^12^ since the increase in HDL-C may be more pronounced in individuals with lower baseline levels. Unfortunately, as there is no baseline HDL-C data from participants in the cohort of Pitanga et al.,^16^ this analysis was not possible. On the other hand, the study brings interesting hypotheses that can be tested in randomized controlled trials.

The study is limited by its observational nature, its cross-sectional design, and the use of self-reported PA questionnaires, which may add some subjectivity to the information. Thus, there is still debate regarding the specific intensity and volume of training for men and women aiming at raising HDL-C.
